# Physiotherapists’ and midwives’ views of increased inter recti abdominis distance and its management in women after childbirth

**DOI:** 10.1186/s12905-020-00907-9

**Published:** 2020-02-27

**Authors:** Catharina Gustavsson, Martin Eriksson-Crommert

**Affiliations:** 1 Center for Clinical Research Dalarna, Uppsala University, Nissers väg 3, S-79182 Falun, Sweden; 2 School of Education, Health and Social Studies, Dalarna University, SE-79188 Falun, Sweden; 3 Department of Public Health and Caring Sciences, Family Medicine and Preventive Medicine, Uppsala University, BMC, Box 564, SE-751 22 Uppsala, Sweden; 4 University Health Care Research Center, Faculty of Medicine and Health, Örebro University, Region Örebro county, Box 1613, 701 16 Örebro, Sweden

**Keywords:** Diastasis recti, Health professionals, Health promotion, Management, Perception, Post-partum health, Primary healthcare, Qualitative research method, Sweden, women’s health

## Abstract

**Background:**

Physiotherapists and midwives in primary healthcare often encounter women with an increased separation between the two rectus abdominis muscle bellies after pregnancy, a so-called increased *inter recti distance* (IRD). There are few studies on the contribution of increased IRD to the explanation of post-partum health complaints, and very little guidance in the literature for health professionals on the management of increased IRD. The aim of this study was to describe how physiotherapists and midwives in primary healthcare perceive the phenomenon of increased IRD and its management in women after childbirth.

**Methods:**

A purposeful sampling approach was used to select physiotherapists and midwives working in primary healthcare in three large county council healthcare organisations in Sweden having experience of encountering women with increased IRD after pregnancy. Sixteen physiotherapists and midwives participated in focus group discussions. Four focus groups with four participants in each were undertaken. A semi-structured topic guide was used to explore responses to the research questions and the discussions were analysed using qualitative content analysis.

**Results:**

We identified an overarching theme: *Ambivalence towards the phenomenon increased IRD and frustration over insufficient professional knowledge*. The theme included three categories: Uncertainty concerning the significance of increased IRD as a causal factor for functional problems; perceived insufficient professional knowledge base for the management of increased IRD; and lack of inter-professional collaboration and teamwork in the management of patients with increased IRD. Due to sparse and somewhat contradictory research findings and absence of clinical guidelines, the health professionals lacked basic preconditions for applying an evidence-based practice concerning increased IRD. They obtained their information about increased IRD from the media and fitness coaches, and hence were somewhat unsure about what to believe regarding the phenomenon.

**Conclusions:**

There was no consensus among the health professionals on how to best approach increased IRD in the clinical setting. Our findings stress the importance of more research to increase the professional knowledge base among physiotherapists and midwives. The findings highlight the urgent need for policies and clinical guidelines advising health professionals in the management of increased IRD and for facilitating inter-professional collaboration and teamwork.

## Background

During pregnancy, the expanding uterus/abdomen changes the geometry of the abdominal muscles. Besides elongation of the rectus abdominis muscles, it may lead to stretching of the connective tissue, linea alba, in-between the two rectus abdominis muscle bellies, resulting in an enlargement of the distance between the medial borders of the muscles, a so-called *increased inter recti distance* (IRD) [1]. The increased separation is often visible as a bulging of the middle line of the stomach while performing movements that put strain on the front of the abdominal wall and front-loading exercises such as sit-ups and abdominal crunches [2, 3].

The reported prevalence of increased IRD varies between studies in part due to different IRD cut-off values for the diagnosis and differences in measurement methods [4]. Early studies have reported that increased IRD affects between 30 and 70% of pregnant women [5], and that the increased IRD may persist in the early postpartum period in about 35–60% of women [6]. A more recent study reported that the prevalence of increased IRD was 100% in late pregnancy and decreased to 39% at 6 months postpartum [7]. Another study found a prevalence of increased IRD of 60% at 6-weeks postpartum, 45% at 6 months postpartum, and 33% at 12 months postpartum [8]. Increased IRD has also been found in 39% of older parous women [9], and in 52% of women in a menopausal patient population [10], which indicate that IRD may persist past childbearing years.

Physiotherapists and midwives working in primary healthcare are often the ones that encounter women having an increased IRD after pregnancy. As health professionals, they are supposed to adhere to evidence-based knowledge and implement an evidence-based practice [11, 12]. According to the Promoting Action on Research Implementation in Health Services (PARIHS) framework, implementation of evidence-based practice is a function of the interaction between the degree of scientific support (*evidence*), the healthcare environment (*context*), and how the implementation of evidence-based practice is supported (*facilitation*) [13, 14]. However, there are few studies investigating the contribution of increased IRD to the explanation of post-partum health complaints, such as lumbo-pelvic pain, pelvic-floor dysfunction and impaired abdominal muscle strength [7, 8, 10, 15–20], and in addition, these studies show partly contradictory results. A recent review describes an association between IRD width and health-related quality of life, abdominal muscle strength and severity of low back pain [17], but the level of evidence is low. Thus, there is very little guidance in the literature for health professionals to support the management of increased IRD in women after pregnancy. In recent decades, many health professionals and fitness coaches have applied approaches that focus on rebuilding the inner core and/or strengthening the pelvic floor by training the transverse abdominal muscle and pelvic floor muscles, in order to close the increased muscle separation and reduce functional symptoms [21–23]. However, recent research suggests that such training does not affect the width of the increased IRD [24] and that it is not correlated with either pelvic floor dysfunction [25] or low back pain [15].

To the best of our knowledge, no studies have investigated the experiences of healthcare professionals who encounter women with increased IRD after childbirth. The aim of this study was to describe how physiotherapists and midwives in primary healthcare perceive the phenomenon of increased IRD and its management in women after childbirth.

## Materials and methods

### Study design

This study had a descriptive study design. The study used qualitative methods for content analysis [26] with an inductive approach [27] to analyse focus group data.

### Participants

We used a purposeful sampling approach [28] to select physiotherapists and midwives in primary healthcare in three county council healthcare organisations in Sweden having experience of encountering women with increased IRD after pregnancy. Information about the study was distributed by e-mail to all primary care physiotherapists and midwives in the county councils. Those interested in participating responded by e-mail, and were contacted by the researchers. The sampling aimed to represent variation in type of profession (i.e. physiotherapists and midwives) and possible variation in the organisation of healthcare services in each county council. For example, in one of the county councils some of the physiotherapists were involved in the parental groups held by the midwives, but in the other two they were not involved.

The participants consisted of 16 health professionals working in primary healthcare: seven midwives and nine physiotherapists. They were divided into four focus groups with four participants in each. The groups were composed to ensure participation of both physiotherapists and midwives in each group. For practical reasons (i.e. geographical closeness), each focus group consisted of participants from one county council healthcare organisation. Each focus group met once. The background characteristics of the participants are provided in Table 1.

**Table 1:** Background characteristics of physiotherapists and midwives participating in the focus groups (*n* = 16)

Focus group number	Person ID-code	Profession	Years of professional experience	Healthcare region
Focus group 1	FG1–1, PT	Physiotherapist	31 years	A
	FG1–2, PT	Physiotherapist	15 years	A
	FG1–3, MW	Midwife	27 years	A
	FG1–4, MW	Midwife	36 years	A
Focus group 2	FG2–1, MW	Midwife	29 years	A
	FG2–2, PT	Physiotherapist	14 years	A
	FG2–3, PT	Physiotherapist	12 years	A
	FG2–4, PT	Physiotherapist	2 years	A
Focus group 3	FG3–1, MW	Midwife	27 years	B
	FG3–2, MW	Midwife	19 years	B
	FG3–3, PT	Physiotherapist	5 years	B
	FG3–4, PT	Physiotherapist	4 years	B
Focus group 4	FG4–1, MW	Midwife	35 years	C
	FG4–2, MW	Midwife	15 years	C
	FG4–3, PT	Physiotherapist	23 years	C
	FG4–4, PT	Physiotherapist	13 years	C

The study was approved by the Regional Ethics Review Board at Uppsala University (No. 2017–316). Verbal and written information about the study was provided to the participants, and written consent was obtained prior to participation in the discussions in the focus groups.

### Procedure for data collection

The focus group format for data collection was chosen to promote interaction and information exchange between the participants. Interaction allows the participants to respond to and build on the reactions of other participants in the group [29]. Group interaction can reveal points of agreement, conflict, and uncertainty. This kind of interaction would have the ability to disclose aspects of understanding which often remain untouched by other research methods such as in-depth interviewing method [30]. The focus group discussions were undertaken during the spring of 2018, and used a semi-structured topic guide [28] containing open-ended questions designed to respond to the research questions of the study:

- What attitudes do physiotherapists and midwives have to the phenomenon of increased IRD and its management in women after childbirth?
- How do physiotherapists and midwives perceive their knowledge and competence in encountering, managing, and treating women with increased IRD?

During the focus groups, the moderator posed probing and follow-up questions, restated and summarised information, and asked the participants to confirm the accuracy of the spoken data [31]. The moderator took actions to ensure that all participants could participate actively and freely in the discussions, i.e. that not one or some of the participants dominated the discussions and held the others back. The session lasted until the moderator sensed that the discussion had reached saturation; that is, when additional information no longer generated new understanding [30]. The focus group sessions lasted for 50–70 min and were carried out in small meeting rooms at the primary healthcare centres. Only the participants, the moderator and a note-taker were present, and they were undisturbed by others. The second author acted as moderator at all of the focus group discussions. The discussions were audio-recorded, and transcribed verbatim by a research assistant directly after each discussion.

### Data analysis

The text files from the focus group discussions were analysed using qualitative content analysis [26] with an inductive approach [27]. The analysis was undertaken by the authors (both of whom are physiotherapists) and two midwifery students. First, the text files were read through in order to identify, condense, and code the meaning units; i.e. specific units of text relating to the research questions. The codes were then grouped together into higher-order subcategories and categories. In order to validate the interpretation, the authors and the midwifery students re-read the text files, discussed the coding, reviewed the preliminary interpretation, and revised the interpretation until consensus was achieved. The analysis was done computerised by coding of the text in Microsoft Word text files and by grouping into subcategories and categories in Microsoft Excel files (Microsoft Office 2016).

## Results

The content analysis of the focus group discussions identified several problems and insufficiencies in the management of increased IRD after childbirth, and generated the overarching theme: *Ambivalence towards the phenomenon increased IRD and frustration over insufficient professional knowledge*. The theme included three categories: Uncertainty concerning the significance of increased IRD as a causal factor for functional problems; perceived insufficient professional knowledge base for the management of increased IRD; and lack of inter-professional collaboration and teamwork in the management of patients with increased IRD. The categories and subcategories are outlined in Table 2 and described in further detail below, illustrated with quotations from the focus groups in italics.

**Table 2:** Qualitative content analysis of experiences of healthcare consultations with women having increased IRD after childbirth among physiotherapists and midwives working in primary healthcare (*n* = 16), and how the health professionals experienced the management of these women

Overall theme: Ambivalence toward the phenomenon increased IRD and frustration over insufficient professional knowledge	
Categories	Subcategories
Uncertainty concerning the significance of increased IRD as a causal factor for functional problems	Unsure about the relevance of increased IRD as a clinical health problem
	Indecisiveness about whether the increased media focus on increased IRD had helped to highlight an important women’s health issue or had faultily made a health problem of something essentially normal
Perceived insufficient professional knowledge base for the management of increased IRD	Lack of education during physiotherapy and midwife university studies about the management of increased IRD
	A perceived lack of concern from the central healthcare organisation in extending the knowledge base for the management of increased IRD by clinical guidelines and education
	Dependency on possibly non-evidence based information conveyed by the media and commercial fitness coaches for the management of increased IRD
Lack of inter-professional collaboration and teamwork in the management of patients with increased IRD	Perceived differences between physiotherapists and midwives in regard to professional knowledge and competence emphasised the need for extended inter-professional collaboration
	Need for local routines describing teamwork, referral pathways and when/who to refer to special competences

IRD Inter recti distance

### Uncertainty concerning the significance of increased IRD as a causal factor for functional problems

There was no consensus among the participants on whether increased IRD should be perceived as a condition that require medical attention. Most of the participants were unsure about and lacked knowledge regarding the relevance of increased IRD as a clinical health problem. Those that did regard increased IRD as a clinical health problem had different views about whether it primarily is a psychosocial or a functional problem. Several of the midwives regarded increased IRD as a psychosocial problem, with the main issue being the women’s concerns about their changed physical appearance and worries about worsening the condition. Most of the physiotherapists regarded increased IRD as a functional problem often observed along with insufficient pelvic floor control and insufficient core muscle function. However, the physiotherapists were not sure whether increased IRD was a causal factor in functional problems or a factor otherwise associated with the functional problems.

*“I’m also wondering a bit about the connection between the diastasis and functional problems. Because I think there must be women who have an increased diastasis but no symptoms. If that’s the case, then you can’t always draw a line between diastasis and functional problems. But I don’t know. I don’t know enough about it. What if it’s like when you x-ray someone’s back and you see a lot of bad things even though the person doesn’t have any pain? I wonder, does it correlate? I don’t know. It would have been good to know.” (FG3–4, PT)*

The physiotherapists reported that women rarely sought help due to an increased IRD per se, but usually due to low back and pelvic pain; the increased IRD was then mentioned during the medical history or identified during the physical examination. All the physiotherapists except one regarded the phenomenon of increased IRD as important to address in patient consultations. Several of the participants pointed out that the increased media focus in recent years had raised awareness about IRD. Some claimed that this increased media focus had helped to highlight an important women’s health issue, but several also felt there was a potential risk of making something essentially normal into a health problem.

*“On the one hand, it’s a good thing that it’s growing, that there’s more awareness of women’s health issues. The downside is that we’re making a problem out of what is essentially normal. And that’s the hardest thing to balance. I feel that sometimes we problematize things that are normal parts of having a baby. You aren’t a robot, and you won’t be the same as before. I feel that it’s hard to handle this type of demand on the healthcare organization: ‘Fix this now’, ‘I want to be the way I was before’. That’s the hardest part, that they believe everything will be as usual afterwards. There are strong demands put on us to respond to that.” (FG3–1, MW)*

### Perceived insufficient professional knowledge base for the management of increased IRD

The participants reported that the recent media focus on increased IRD after childbirth meant that more women were asking health professionals about increased IRD, which had subsequently increased the need among health professionals for more knowledge on how to manage increased IRD. None of the participants had gained knowledge about increased IRD during their professional education (undergraduate studies), and none had any other courses or additional education arranged by their county councils. All they knew about IRD was based on personal interest in the subject and on what they had drawn from their clinical experiences of patient encounters. All of the participants expressed an urgent need for more education and evidence-based knowledge. Those of the participants who had tried to search for scientific literature claimed that scientific studies and evidence-based information on IRD were both very scarce.

*“We don’t have the knowledge even though it’s important since patients ask us about it. The patients ask: ‘What can I do?’ And I don’t have enough knowledge. I would like to have that knowledge.” (FG1–4, MW)*

*“We don’t have the knowledge. It wasn’t mentioned at all during my physiotherapy education. And I think it’s the same for midwives. I know more than my colleagues, but that’s because I’ve chosen to search for knowledge. I read about it, because I’m interested in the subject.” (FG2-3 (PT)*

*“I try to make time, my own personal time, to Google and search for articles. But I find embarrassingly little about physiotherapy with regard to increased diastasis recti.” (FG2–4, PT)*

The participants also expressed a need for clinical guidelines and tailoring of guidelines to fit their own healthcare organisations. They perceived that the county councils they worked in lacked concern about extending the knowledge base for the management of increased IRD to the health professionals, for example by providing courses, clinical guidelines, and tailored local work routines.

*“There’s no collated knowledge about it. We need some sort of national clinical guidelines outlining how we should do it.” ... “It should be one and the same for the whole of Sweden, not just for the healthcare region. Then every patient would get the same management.” (FG2–4, PT)*

*“If it came from higher up* [i.e. from management level in the healthcare organisation]*, stating that this is something we should do, then we’d all have to do it. But now, you have to push the issue yourself.” (FG2-3, PT) “Exactly!” (FG2-1, MW) “The healthcare organisation hasn’t been interested in this. No-one above us* [i.e. at management level in the healthcare organisation] *has been interested in allocating resources to this question.” (FG2–2, PT)*

This dearth of evidence-based knowledge meant that the participants had to rely on information from the media and commercial fitness coaches for the management of increased IRD. When searching for knowledge, they found the same information and from the same sources as the women seeking their help. The participants considered some of these information sources to be reliable, but they also had hesitations about the value and evidence base for some of the information. Mainly, they questioned the reliability of some of the information due to obvious commercial interests.

*“I’ve been looking for information, and there are so many general opinions on managing diastasis recti, but I don’t find many professional opinions. I was in the bookshop yesterday and saw that AA, the personal trainer from the TV, and BB, the media personality, had written a book on ‘fitness for mums’. I had a bit of a look through the book and thought: is this correct, do they really know this for sure, is this evidence-based?” (FG3-1, MW) “I also wonder if they really do know? I don’t listen to it that much, because I’m sceptical about its being supported by evidence.” (FG3-2, MW) “Exactly, have they, like, just made something up?” (FG3-1, MW) “Isn’t AA just a personal trainer? I don’t think she’s got any other qualifications.” (FG3–3, PT)*

This perception of insufficient evidence-based professional knowledge meant that the participants were unsure of how to approach and treat women with increased IRD.

*“I think it’s very hard to know how to think about this* [i.e. increased IRD]*. We’ve been doing core stabilisation exercises for the low backs for a long time, but if that also goes for the diastasis recti, I’m not sure. Sometimes I feel I’m walking on thin ice: how should I value the physical findings, who should do specific exercises, what’s normal?” (FG1-2, PT).*

*“It feels as if whatever you’re doing is ‘home-made’.” (FG3–2, MW)*

*“What can I hold on to? It’s hard to know what to do and what to think.” (FG4-4, PT) “When I stand there in the room, I try to find the way, in every specific case I try to find the way. It’s kind of a wide subject. Maybe there’s no consensus. Maybe that’s why it sometimes seems a bit indecisive.” (FG4–3, PT)*

### Lack of inter-professional collaboration and teamwork in the management of patients with increased IRD

Most of the interviewed health professionals felt that there should be more emphasis on managing increased IRD. In particular, there was a need for an extended and improved inter-professional collaboration between physiotherapists and midwives in primary healthcare, given their perceived differences and ability to complement each other with regard to professional knowledge and competence. The midwives did not ask women about increased IRD as part of their routine care after childbirth; when increased IRD was brought up in patient encounters, it was always on the woman’s initiative. The midwives perceived that increased IRD was not within their professional area, and expressed a need for increased cooperation with a physiotherapist. Some midwives suggested that they would like to be able to provide women with pre-printed advice on lifestyle and exercises. All the midwives expressed the wish to have someone to refer the woman to for further management. The physiotherapists reported that it was rare for women to be referred to them primarily for assessment and management of increased IRD; usually they discovered increased IRD among women referred to them for other reasons, such as low back or pelvic pain. They welcomed a more explicit teamwork with midwives, suggesting that physiotherapists could be a part of the healthcare team in the parental-preparation groups that soon-to-be parents are offered before the birth of their first child. All participants acknowledged that local guidelines describing referral pathways, including guidance on when and who to refer to, would facilitate teamwork and inter-professional collaboration.

*“The most difficult part isn’t having to say that I don’t have the knowledge. The most difficult part is not having anyone to refer these patients to, someone who I know does have the knowledge.” (FG3-3, PT) “What do I do then? Because I really want to be able to refer these women to someone who can help them.” (FG3–2, MW)*

*“I want to be able to refer to the right professional, a physiotherapist or a maternal fitness expert. That’s my role in this; to note and pay attention to the diastasis recti, talk to the woman about it, and then refer her to an expert. Because I’m not the expert in these cases.” (FG4-1, MW) “I believe it would be very enriching if we* [i.e. physiotherapists and midwives] *were to cooperate more: we could learn a lot from each other’s expertise.” (FG4–3, PT)*

## Discussion

To our knowledge, this is the first study investigating how health professionals who encounter women with increased IRD after childbirth perceive the management of these women. The findings revealed that the health professionals perceive that they have insufficient knowledge about increased IRD, which they believe is problematic since it leads to inadequate management of increased IRD after childbirth. The findings generated the overarching theme: *Ambivalence towards the phenomenon increased IRD and frustration over insufficient professional knowledge*. In summary, the results show that, due to sparse and somewhat contradictory research findings and the absence of clinical guidelines, these health professionals perceived that they lacked basic preconditions for applying an evidence-based practice concerning increased IRD. The health professionals expressed that they obtained their information about increased IRD from the media and fitness coaches, and hence were somewhat unsure about what to believe regarding the phenomenon and how to handle/manage women with increased IRD in their clinical practice. The health professionals’ uncertainty about the clinical importance of increased IRD largely mirrors the available research literature to date, which is very limited and somewhat confusing. For example, increased IRD has been reported to be associated with pain in the abdominal and pelvic area [19], but not with low back pain [8, 19]. However, in clinical practice it is not always easy to discriminate between pain originating from the low back versus pelvic area, which make matters difficult for health professionals who seek more information about the condition.

All but one of the participants in the focus groups expressed that they were unsure about and perceived that they lacked knowledge regarding the phenomenon of increased IRD and its management in women after childbirth. They had hesitations concerning the relevance of increased IRD as a clinical health problem, and whether it should be seen primarily as a psychosocial or a functional problem. However, although they were not unanimous regarding the extent to which increased IRD was a functional problem, all but one of the participants considered the phenomenon important to address regardless, since more and more women are seeking help for it. This line of reasoning can also be found in the scientific literature, where authors have suggested that it is better to treat increased IRD conservatively than not at all, despite the unclear evidence base [19].

The participants perceived that they did not have enough knowledge and competence in managing women with increased IRD after childbirth. They reported that they obtained their information from the same websites that they recommended to their patients. However, most of their patients had read this information beforehand, creating a situation where the patient knew as much, or sometimes more, than the health professional from whom they sought advice. In particular, the participants communicated a need for clinical guidelines and education providing evidence-based knowledge. The participants called for support from the healthcare organisation in increasing the knowledge base and actively providing explicit clinical guidelines including referral pathways; that is, when and whom to refer to. In the absence of clinical guidelines, it is unsurprising that health professionals feel unsure and become strongly influenced by the opinion of the woman seeking help. However, although the participants asked for guidelines, previous studies from within the field of physiotherapy show that clinical guidelines do not always have an impact on clinical management [32, 33]. It has also been shown that even when physiotherapists know about the existence of clinical guidelines, they are not always familiar with the content [34]. The implication of all this is that despite the slim state of the literature, the phenomenon of increased IRD after childbirth should be included in undergraduate programs in physiotherapy and midwifery, in order to give these healthcare professionals an awareness of and the ability to address this phenomenon when they begin clinical practice. Furthermore, all the health professionals in this study undisputedly acknowledged the importance of inter-professional collaboration between physiotherapists and midwives in primary healthcare in the management of patients with increased IRD. The organisation of such collaboration and the structuring of referral pathways should be possible to achieve even in situations when evidence-based knowledge of the management of the condition as such is insufficient or lacking.

### Strengths and limitations

By using a qualitative method for data collection, a broad range of factors affecting the management of increased IRD post-partum were revealed that would otherwise have been difficult to identify through a quantitative design. Data was collected through focus groups instead of individual interviews since focus groups were deemed more suitable to promote interaction and information exchange between participants. Indeed, all participants contributed actively and freely in the discussions. The discussions involved high degree of interaction between participants and reflections that built on statements from others, which generated a very rich data material. A purposeful sampling strategy was used in order to present maximum variation and contribute to obtaining a rich and broad material, as recommended in the literature [35]. The sample was selected to include variation in those participant characteristics and settings that were believed to have relevance to the conceptualisation of the phenomenon. However, this method carries the risk of selection bias or an incomplete sample. In this study, credibility and transferability were enhanced by using a sample of participants representing relevant professional backgrounds in primary healthcare in three different county councils. Overall, there were similar experiences across focus groups regardless of county council, which increases the credibility of the results. Still, the small number of participants limits transferability. It is possible that the participants were not representative of all people with the same professional background. For example, since the health professionals volunteered to participate in the focus groups, it is likely that they were more interested in and had more knowledge about the subject than most of their colleagues. However, they still regarded their knowledge as insufficient. Thus, knowledge about the management of increased IRD is probably even lower in the whole population of physiotherapists and midwives in primary healthcare. To the best of our knowledge, there are no previous studies similar to the present one, and so we cannot compare our findings of the health professionals’ experiences with findings in other studies. Moreover, we only interviewed stakeholders within the healthcare organisations, and so the perspective of the patient was not included. However, the patient perspective is an important aspect. Thus, we are underway of undertaking a separate study investigating the views of women with increased IRD postpartum by in-depth interviews. To reduce the risk of bias in data collection and analysis, respondent validation was undertaken during the focus group discussions [31]; i.e. the moderator posed probing and follow-up questions, restated and summarised information, and asked the participants to confirm the accuracy of the spoken data. To increase trustworthiness in the interpretation of the data, both researchers, along with two midwifery students, read the text files of the interviews and participated in discussing the coding, categorisation, and interpretation [31, 36].

## Conclusions

There was no consensus among the health professionals on how to best approach increased IRD in the clinical setting. Our findings stress the importance of more research to increase the professional knowledge base among physiotherapists and midwives. The findings highlight the urgent need for policies and clinical guidelines advising health professionals in the management of increased IRD and for facilitating inter-professional collaboration and teamwork.

## Data Availability

The datasets generated and analysed during the current study are not publicly available as they consist of quotes by the interview subjects that might contain information, which could reveal the identity of individuals. But datasets are available from the corresponding author upon reasonable request.

## Abbreviation

IRD: Inter Recti Distance
